# Special Issue “Molecular Advances in Cancer Immunotherapy”

**DOI:** 10.3390/ijms262210839

**Published:** 2025-11-08

**Authors:** Niels Schaft

**Affiliations:** 1 Department of Dermatology, Universitätsklinikum Erlangen, Friedrich-Alexander-Universität Erlangen-Nürnberg, 91054 Erlangen, Germany; niels.schaft@uk-erlangen.de; 2 Comprehensive Cancer Center Erlangen European Metropolitan Area of Nuremberg (CCC ER-EMN), 91054 Erlangen, Germany; 3 Deutsches Zentrum Immuntherapie (DZI), 91054 Erlangen, Germany; 4 Bavarian Cancer Research Center (BZKF), 91054 Erlangen, Germany; 5 CCC-WERA, 91085 Erlangen, Germany

Cancer immunotherapy is defined as a type of cancer treatment that helps the immune system recognize and attack cancer cells more effectively. Instead of targeting the cancer cells directly (like chemotherapy or radiation), immunotherapy boosts or modifies the body’s natural defenses to fight cancer. Despite its relatively simple definition, immunotherapy encompasses a plethora of therapeutic possibilities ranging from cytokine treatments to antibody therapies, checkpoint inhibitors [1], cancer vaccines, adoptive (T-) cell therapies [2,3,4,5], CAR-cell therapies [6,7,8], and other therapies [9,10], or combinations thereof [11,12,13].

Although cancer immunotherapy has been proven to be effective in patients with a broad variety of hematological and solid malignancies [14], there is increasing evidence that its efficacy varies [15]. The therapies themselves should be improved, and investigations should be conducted on the negative influence of the tumor microenvironment (TME) [16,17,18], toxicity management, adverse immune-related side effects [19,20,21], response prediction, and the use of immunotherapy in special patient populations. This Special Issue presents a valuable collection of papers on molecular advances in cancer immunotherapy with a view to understanding how novel and/or improved immunotherapies can be developed for cancer treatment; it includes up-to-date reviews, original research articles, a communication, and a perspective, addressing a wide range of subjects—from non-coding RNAs in cancer cells to glycan structures involved in osteosarcoma.

The first review [22] gives an overview of the current development and applicability of biomarkers for immune checkpoint inhibitor responses in non-small-cell lung cancer (NSCLC). At present, programmed death-ligand 1 (PD-L1) expression is the only validated biomarker used to predict responses to immune checkpoint inhibitors (ICIs) [23]. However, its reliability and predictive accuracy are often inconsistent [24]. This review highlights emerging biomarkers that are either in development or undergoing validation, which show potential for better distinguishing between patients who are likely to respond to ICI therapy and those who are not. As summarized in Figure 1, several categories of biomarkers are discussed: (1) PD-L1 expression is analyzed in multiple ways as a biomarker for NSCLC, namely (a) PD-L1 expression on tumor cells, (b) PD-L1 expression on circulating tumor cells, (c) plasma soluble PD-L1 (sPD-L1), and (d) PD-L1 polymorphisms (Figure 1 and [22]). (2) Gene-expression-based biomarkers [25] include (a) *HNRNPA2B1*, *IGF2BP2*, *NSUN4*, and *ALYREF*; (b) immune-score; (c) *KAT2B*; (d) *MAP1A/1B/1S/4/6/7D1/7D3*; and (e) *TCR* co-expression (Figure 1 and [22]). (3) Tumor mutation burden (TMB; non-synonymous mutations in the coding regions of the tumor genome [26]) is analyzed. (4) Complete-blood-count (CBC)-based biomarkers [27] include (a) absolute eosinophil count (AEC), (b) absolute lymphocyte count (ALC), (c) absolute neutrophil count (ANC), (d) ANC:ALC ratio, (e) derived neutrophil-to-lymphocyte ratio (dNLR), (f) hemoglobin (HGB), (g) myeloid-to-lymphocyte ratio (M:L), (h) monocyte-to-lymphocyte ratio (MLR), (i) neutrophil-to-lymphocyte ratio (NLR), and (j) platelet-to-lymphocyte ratio (PLR) (Figure 1 and [22]). (5) Peripheral blood mononuclear cell (PBMC)-based biomarkers [28] include (a) CCR7^−^ CD45RA^−^ CD8^+^ T cells, (b) (FoxP3^+^) Treg cells, (c) granulocytic myeloid-derived suppressor cells (Gr-MDSC), (d) PD-1^+^ CD8 T cells, (e) PD-L1^+^ CD14^+^ monocytes, (f) TIGIT^+^ T cells, (g) TIM-3^+^ T cells, and (h) the ratio of Tregs to Lox-1^+^ PMN-MDSCs (TMR) (Figure 1 and [22]). (6) Tumor-infiltrating-immune-cell-based biomarkers [29] include (a) CD8^+^ effectors in TILs, (b) TIL distribution, and (c) TIL levels (Figure 1 and [22]). (7) Extracellular-vesicles [30] are analyzed in multiple ways as biomarker for NSCLC, namely (a) CD41a^−^/CD31^+^/CD45^−^ EVs, (b) EV-miR-625-5p, (c) PD-L1 EVs, (d) PD-1/PD-L1 protein and mRNA EVs, (e) tetraspanin EVs (CD9, CD81, CD63), and (f) TGF-β EVs (Figure 1 and [22]). (8) Imaging-based biomarkers include positron emission tomography–computed tomography (PET-CT)[31,32] with (a) ^18^F-FDG to determine the metabolic tumor volume (tMTV), (b) ^89^Zr-atezolizumab, and (c) ^89^Zr-durvalumab (Figure 1 and [22]). Finally, (9) the lung microbiome can be used as a biomarker (Figure 1 and [22,33]).

In conclusion, alongside PD-L1 expression, a plethora of new biomarker candidates are tested and validated to more accurately predict responses to ICI treatment in NSCLC patients.

In the second review, Binder et al. [34] describe a small part of existing RNAs, namely non-coding (nc)RNAs, which perform a range of intracellular regulatory roles. This review highlights small interfering RNA (siRNA) and microRNA (miRNA), which are molecules that act like molecular switches, fine-tuning how genes are turned on or off by influencing how mRNA is translated into proteins. They are essential for the normal functioning of all cells in the human body, including cancer cells [35,36].

In cancer research, scientists are especially interested in targeting two key players: cancer cells, which grow uncontrollably, and immune cells, which often fail to fight them effectively. Understanding how siRNAs and miRNAs regulate cell behavior could open the door to new kinds of treatments. By using these molecules to “reprogram” cancer cells or boost the immune system’s ability to recognize and destroy tumors, researchers hope to develop smarter, more precise cancer therapies. The first part of the review presented in [34] explores what siRNAs and miRNAs do and how new RNA-based technologies work. The second part looks at how these innovations might be used to treat cancer—particularly by influencing immune cells in the tumor environment, such as tumor-associated myeloid cells.

Myeloid cells within the TME play a dual role in anti-tumor immunity. These cells are key regulators of tumor-specific immune responses and are found in large numbers within tumors, making their proper pro-inflammatory activity essential for mounting an effective defense against cancer. Modifying the immune response can be accomplished by reprogramming these myeloid cells in vivo using siRNA- or miRNA-based therapies, as demonstrated in mouse models [34]. Nevertheless, additional studies are needed to better understand the underlying signaling pathways and intricate regulatory networks that control their behavior.

In their perspective, Waaga-Gasser and Böldicke [37] provide an overview of genetically engineered T cells and recombinant antibodies with the potential to target intracellular neo-antigens [38]. Several T-cell-based cellular therapies directed against neo-antigens are listed, namely T cells that express either (a) a recombinant full-length T-cell receptor (TCR) (TCR-T cells), (b) a chimeric antigen receptor (CAR) incorporating the variable regions of a neo-epitope-specific TCR as the binding domain (TCR-CAR-T cells), (c) a TCR-like antibody serving as the binding domain (TCR-like CAR-T cells), (d) a synthetic T-cell receptor and antigen receptor (STAR), or (e) a heterodimeric TCR-like CAR (T-CAR). The latter two are designed as dual-chain, TCRαβ-based receptors in which immunoglobulin variable regions (VH and VL) are fused to TCR constant domains (TCR-Cα and TCR-Cβ), enabling TCR-like signaling [37].

Furthermore, several recombinant antibodies with the potential to target intracellular neo-antigens, such as (a) TCR-like antibodies, (b) bi-specific antibodies, and (c) intrabodies, are described in [37]. Both T-cell- and antibody-based strategies have advantages and disadvantages, with specific clinical implications and therapeutic limitations [37], but together form a plethora of treatment possibilities that have now been tested in vitro, in vivo, and even in early-phase clinical trials.

Nevertheless, several challenges must be taken into account, including the loss or alteration of neo-antigens, changes in antigen presentation, tumor heterogeneity, and the immunosuppressive characteristics of the tumor microenvironment.

In their communication, Qin et al. describe the sequential use of IFN-α and anti-PD1 antibodies to prevent the recurrence of hepatitis B virus (HBV)-related hepatocellular carcinoma (HCC) [39]. The authors state that it is plausible that anti-PD1 antibodies help prevent cancer recurrence in patients with HBV-related HCC following surgical resection. Nonetheless, the adverse effects linked to anti-PD1 therapy at standard clinical doses, along with the potential risk of HBV reactivation from the remaining viral genome, may limit its feasibility as a standalone preventive strategy against HCC development. Furthermore, it is known that IFN-α therapy exerts both anti-cancer and anti-HBV activities, which might amplify the anti-tumor effects of anti-PD1 treatment and may reduce the dose of anti-PD1 required to prevent HCC recurrence. The authors hypothesized that a sequential combination approach could help eliminate residual or newly emerging tumor cells associated with HBV infection in patients with HBV-related HCC following curative surgery, which they confirmed in both a mouse model of HBV-related HCC, and a clinical phase I trial using PEGylated IFN-α and anti-PD1 antibodies in patients with HBV-related HCC after curative surgery, resulting in HBV suppression or even clearance with mild or moderate adverse events [39].

The mechanisms by which this treatment works can be divided in two parts (Figure 2).

(1) The IFN-α treatment results in (a) an anti-proliferative effect on tumor cells; (b) an immunostimulatory effect on NK and T cells; (c) inhibition of HBV; (d) an increase in CCL4 secretion by tumor cells, resulting in the attraction of CD8^+^ T cells to the TME, the activity of which is increased by the anti-PD1 treatment; and (e) a high-glucose microenvironment, which results in an increase in CD27^+^/CD8^+^ T effector cells, the activity of which is again increased by the anti-PD1 treatment (Figure 2 and [39]). (2) The anti-PD1 treatment results in (a) a decrease in hepatitis B surface antigen (HBsAg), (b) a reversal of T-cell exhaustion, which inhibits viral infection and cancer cell proliferation; and (c) an enhancement in IFN-γ secretion, causing the proliferation and activation of surrounding PBMCs (Figure 2 and [39]).

Taken together, these results justify further exploration and clinical development of this combination therapy.

Because triple-negative breast cancer (TNBC) typically exhibits more mutations than other breast cancer subtypes, it tends to be more immunogenic [40]. Hence, Heimes et al. [41] hypothesized that tumor-infiltrating plasma cells have a prognostic impact in TNBC patients. To investigate this hypothesis, they analyzed the plasma cell markers CD38 and IgκC via immunohistochemistry on samples of 107 patients. They found that higher tumor infiltration with CD38-positive plasma cells was associated with significantly longer metastasis-free survival (MFS) [41]. Furthermore, CD38 and nodal status were identified as independent prognostic factors. However, in a validation experiment using gene expression data from an independent, publicly available TNBC cohort, the CD38 mRNA expression data did not show a significant effect on MFS [41]. Higher IgκC expression was shown to be associated with a better outcome (longer MFS) [41], but this was not significant for protein expression. However, in the validation experiment, higher IgκC mRNA expression was associated with significantly longer MFS [41]. Additionally, there was a significant correlation between plasma cell infiltration and *BRCA* mutation status [41].

In summary, in this retrospective immunohistochemical study, Heimes et al. demonstrated the prognostic relevance of the humoral immune response in a cohort of 107 patients with early-stage triple-negative breast cancer.

Prasse et al. presented a new format for a chimeric antigen receptor (CAR), including a binding domain not based on an scFv, as is usually the case, but on the C-type lectin domain CD301 [42]. This domain is linked to the CD28 and CD3ζ intracellular signaling domains, and the authors introduced this CAR into the NK cell line NK92 through retroviral transduction. CD301 binds to glycan structures, such as Tn antigen, which is a GalNAc residue bound to serine or threonine. This structure can then be further modified to a sialyl-Tn (STn), also recognized by CD301. The authors found that these glycan structures are expressed by osteosarcoma cells, and their CD301-CAR NK92 cells were able to recognize several antigen-expressing osteosarcoma cell lines. This resulted in IFN-γ production, degranulation (as measured by CD107a), and lysis of the tumor cells (Figure 3 and [42]). Additionally, NK92 weakly expressed TIGIT, and therefore, blocking with an anti-TIGIT antibody only resulted in a slight increase in killing efficiency (Figure 3 and [42]).

In general, lectin-based CD301-CARs in combination with ICI could be a valid strategy for osteosarcoma treatment. However, since CD301 ligands can also be detected on normal tissues, like the gastrointestinal tract, this strategy first needs to be carefully investigated before it can be employed to treat patients.

This Special Issue demonstrates the diversity of cancer immunotherapy, and describes several molecular advances in this area of research, ranging from biomarkers for immune checkpoint inhibitor responses in NSCLC [22] to the therapeutic potential of non-coding RNAs [34]; the use of genetically engineered T cells and recombinant antibodies to target intracellular neo-antigens [37]; the sequential use of IFN-α and anti-PD1 antibodies to prevent the recurrence of HBV-related HCC [39]; the prognostic impact of CD38- and IgκC-positive tumor-infiltrating plasma cells in TNBC [41]; and the use of glycan structures in osteosarcoma as targets for lectin-based chimeric antigen receptor immunotherapy [42].

## Figures and Tables

**Figure 1:**
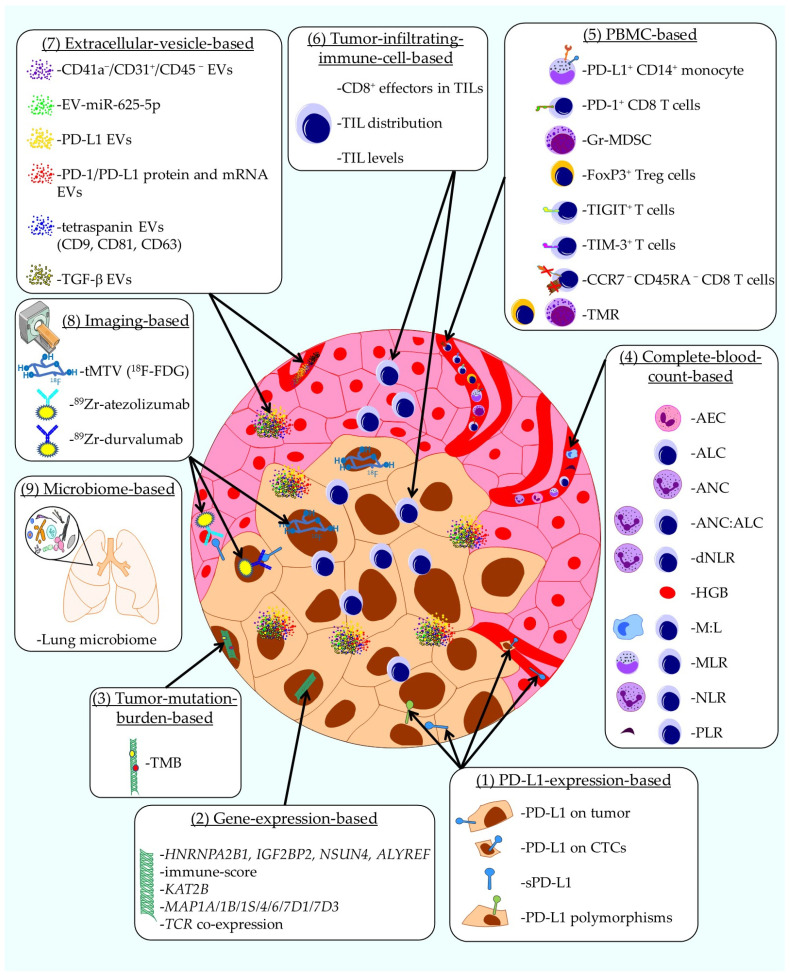
Schematic representation of used and emerging biomarkers for ICI treatment efficiency in NSCLC patients. The categories of biomarkers are as follows: (1) PD-L1 expression, (2) gene-expression-based biomarkers, (3) tumor mutation burden, (4) complete-blood-count (CBC)-based biomarkers, (5) peripheral blood mononuclear cell (PBMC)-based biomarkers, (6) tumor-infiltrating-immune-cell-based biomarkers, (7) extracellular-vesicles, (8) imaging-based biomarkers, and (9) lung microbiome. PD-L1: programmed death ligand 1; NSCLC: non-small-cell lung carcinoma; sPD-L1: soluble PD-L1; TMB: tumor mutation burden; CBC: complete blood count; AEC: absolute eosinophil count; ALC: absolute lymphocyte count; ANC: absolute neutrophil count; dNLR: derived neutrophil-to-lymphocyte ratio; HGB: hemoglobin; M:L: myeloid-to-lymphocyte ratio; MLR: monocyte-to-lymphocyte ratio; NLR: neutrophil-to-lymphocyte ratio; PLR: platelet-to-lymphocyte ratio; PBMC: peripheral blood mononuclear cells; Gr-MDSC: granulocytic myeloid-derived suppressor cells; TMR: ratio of Tregs to Lox-1+ PMN-MDSCs; TILs: tumor-infiltrating lymphocytes; EV: extracellular vesicle; PET-CT: positron emission tomography–computed tomography; tMTV: metabolic tumor volume; CTC: circulating tumor cell. This figure was created with Motifolio.

**Figure 2:**
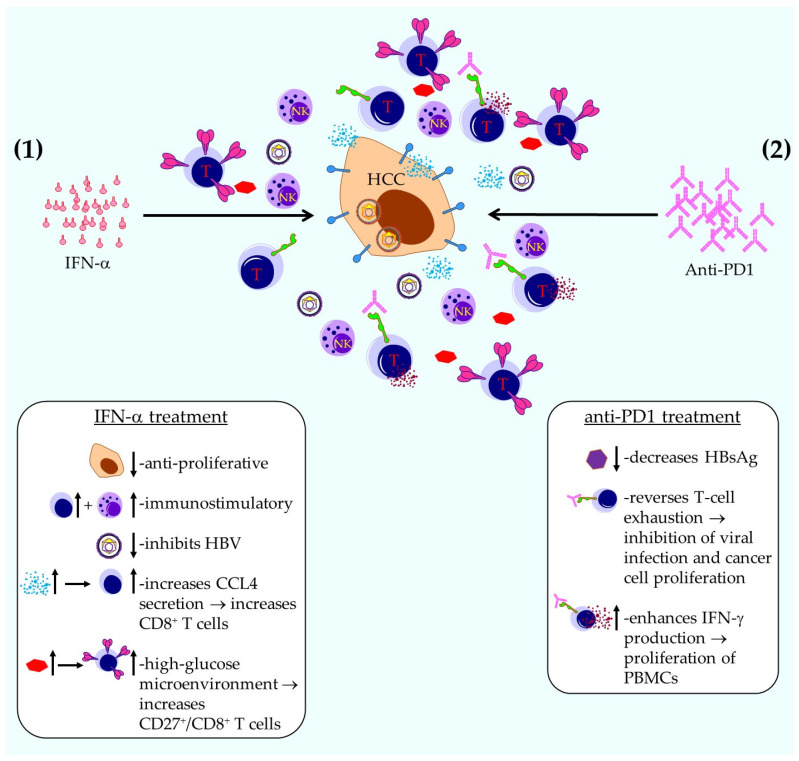
Schematic representation of sequential therapy with IFN-α and anti-PD1 antibodies for inhibiting the recurrence of HBV-related HCC. Shown are possible effects of IFN-α and anti-PD1 treatment. For further explanation see text. This figure was created with Motifolio.

**Figure 3:**
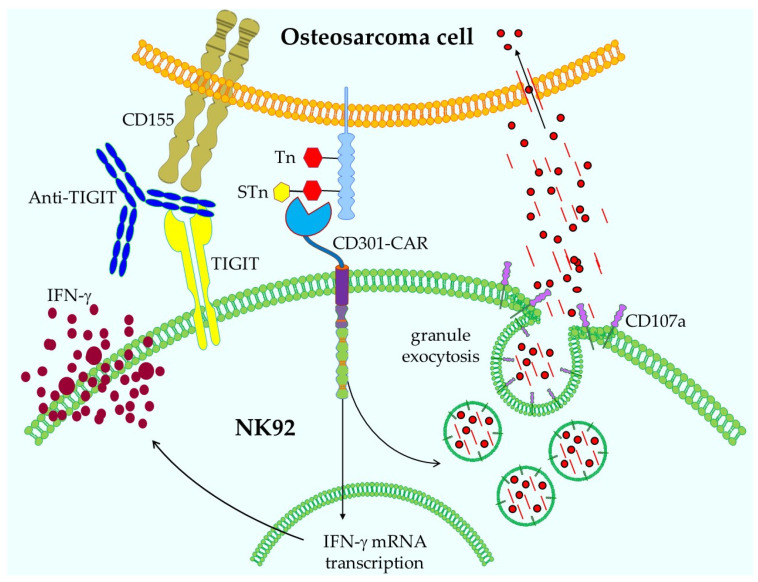
Schematic representation of the functionality of CD301-CAR NK92 cells. After recognition of STn or Tn antigens on osteosarcoma cells by CD301-CAR, the NK92 cells respond with exocytosis of granules loaded with perforin and granzyme B and IFN-γ secretion, thereby killing the tumor cells. Tumor cell lysis can be enhanced by ICI with anti-TIGIT antibodies. Not depicted: the intrinsic lytic mechanisms of NK cells. This figure was created with Motifolio.
